# Reciprocal Interaction between Macrophages and T cells Stimulates IFN-γ and MCP-1 Production in Ang II-induced Cardiac Inflammation and Fibrosis

**DOI:** 10.1371/journal.pone.0035506

**Published:** 2012-05-02

**Authors:** Ya-lei Han, Yu-lin Li, Li-xin Jia, Ji-zhong Cheng, Yong-fen Qi, Hong-jia Zhang, Jie Du

**Affiliations:** 1 Beijing An Zhen Hospital, Capital Medical University, Beijing, China; 2 The Key Laboratory of Remodeling-related Cardiovascular Diseases, Ministry of Education, Institute of Heart Lung and Blood Vessel Diseases, Beijing, China; The Chinese University of Hong Kong, Hong Kong

## Abstract

**Background:**

The inflammatory response plays a critical role in hypertension-induced cardiac remodeling. We aimed to study how interaction among inflammatory cells causes inflammatory responses in the process of hypertensive cardiac fibrosis.

**Methodology/Principal Findings:**

Infusion of angiotensin II (Ang II, 1500 ng/kg/min) in mice rapidly induced the expression of interferon γ (IFN-γ) and leukocytes infiltration into the heart. To determine the role of IFN-γ on cardiac inflammation and remodeling, both wild-type (WT) and IFN-γ-knockout (KO) mice were infused Ang II for 7 days, and were found an equal blood pressure increase. However, knockout of IFN-γ prevented Ang II-induced: 1) infiltration of macrophages and T cells into cardiac tissue; 2) expression of tumor necrosis factor α and monocyte chemoattractant protein 1 (MCP-1), and 3) cardiac fibrosis, including the expression of α-smooth muscle actin and collagen I (all p<0.05). Cultured T cells or macrophages alone expressed very low level of IFN-γ, however, co-culture of T cells and macrophages increased IFN-γ expression by 19.8±0.95 folds (vs. WT macrophage, p<0.001) and 20.9 ± 2.09 folds (vs. WT T cells, p<0.001). In vitro co-culture studies using T cells and macrophages from WT or IFN-γ KO mice demonstrated that T cells were primary source for IFN-γ production. Co-culture of WT macrophages with WT T cells, but not with IFN-γ-knockout T cells, increased IFN-γ production (p<0.01). Moreover, IFN-γ produced by T cells amplified MCP-1 expression in macrophages and stimulated macrophage migration.

**Conclusions/Significance:**

Reciprocal interaction between macrophages and T cells in heart stimulates IFN-γ expression, leading to increased MCP-1 expression in macrophages, which results a forward-feed recruitment of macrophages, thus contributing to Ang II-induced cardiac inflammation and fibrosis.

## Introduction

Hypertension is a multi-factorial chronic inflammatory disease. It induces cardiac remodeling, which is characterized by inflammation, fibrosis and hypertrophy, a major cause of heart failure. In hypertension stage, damaged vasculature release inflammatory signals to recruit leukocytes into cardiac tissues, and then initiate fibrosis cascade [1]. The interaction between these inflammatory cells is complex and is still largely unknown.

The renin-angiotensin system, especially angiotensin II (Ang II), plays a major role in inflammation and cardiac fibrosis [2]. Ang II can directly or indirectly activate different signaling pathways to trigger the inflammatory response and fibrosis in hypertension [3]. Several studies point to a role for the immune system in Ang II–dependent hypertension and its complications. Blockade of inflammatory responses blunted the chronic hypertensive response to Ang II, thus reducing cardiac hypertrophy [4]. Moreover, emerging evidence shows that activated effector T cells do not simply accompany hypertension but rather support a role of inflammation in this disease [5]. Co-stimulation of T cells via B7 ligands was found essential for the development of hypertension [6]. Ang II infusion in rats stimulated a T helper 1 (Th1) immune profile in splenocytes, which could be suppressed by the Ang II type I receptor (AT1R) blocker olmesartan but not by hydralazine, even the two treatments lowered blood pressure to a similar extent [7]. Th1 but not Th2 immune responses were positively associated with both outward vascular remodeling and intimal expansion of ascending thoracic aortic aneurysm [8]. However, the specific role of interaction of T cells and the macrophage on inflammatory response and cardiac fibrosis remains unclear.

IFN-γ is produced by activated T cells, macrophages or dendritic cells [9] and is a potent activator of macrophage and Th1 responses and production of inflammatory cytokines [10]. IFN-γ can augment [11] or suppress [12] autoimmunity and the associated abnormalities in context- and disease-specific manners. Function of different cellular sources of IFN-γ in different types and phases of immune response is multifarious. In renovascular hypertension models, endogenously increased Ang II production induced T-lymphocyte secretion of IFN-γ that induced a switch from stable to vulnerable plaques [13]. Furthermore, the expression of both tumor necrosis factor α (TNF-α) and IFN-γ secreted by T cells was increased in mice with Ang II-induced hypertension [5]. However, the exact role of IFN-γ, such as the cellular sources for its production and its effector cells, in Ang II-induced inflammation and remodeling remains unclear.

In this study, we aimed to study the role of interaction between T cells and macrophages in regulating inflammatory responses in cardiac inflammation and fibrosis induced by Ang II infusion. We found IFN-γ deficiency in mice prevented Ang II-induced inflammatory cells infiltration and cardiac fibrosis. The underlie mechanism involves a reciprocal interaction between T cells and macrophages to stimulate IFN-γ and MCP-1 production in T cells and macrophages respectively, which results a forward-feed recruitment of macrophages.

## Results

### IFN-γ Expression Is Increased in Ang II-infused Hearts

**Figure 1:**
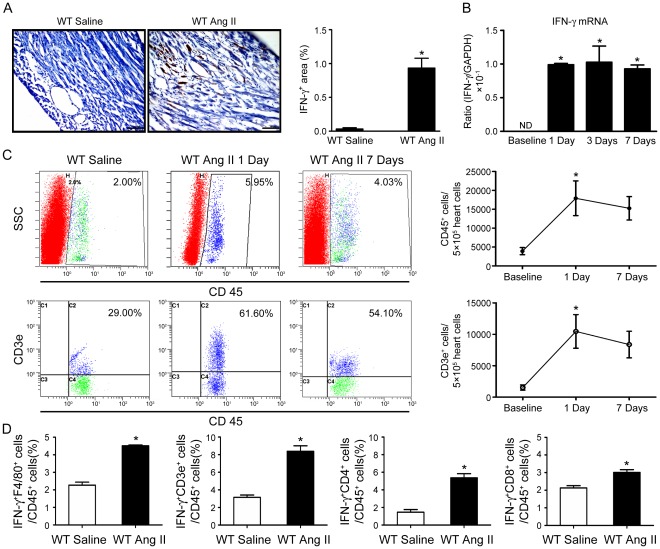
Interferon-γ (IFN-γ) expression is increased and primarily produced by T cells in angiotensin (Ang) II-infused mouse hearts. **A**, Immunohistochemical staining of IFN-γ in wild type (WT) mice after infusion of Ang II (1500 ng/kg/min) or saline for 7 days. Bar graph shows semi-quantitative analysis of IFN-γ^+^ cells (*n* = 4). Magnification: ×200. **B**, Real-time PCR analysis of IFN-γ mRNA expression in WT mice after saline or Ang II infusion for 1, 3 and 7 days. Data are mean±SEM. * *p*<0.05 versus WT saline control. **C**, Inflammatory cells infiltrated very early after Ang II infusion in WT mice. Flow cytometry analysis of (*top panel*) CD45^+^, (*bottom panel*) CD3e^+^ cells infiltration of control and Ang II-treated WT at day 1 and day 7. Results are expressed as the number of CD45^+^ and CD3e^+^ cells in 5×10^5^ heart cells and represent the means±SEM (*right panel*; *n* = 4). **D**, T cells are primary sources for IFN-γ production. Showed are bar graphs of flow cytometry analysis of cell source of IFN-γ in Ang II-treated hearts using antibodies against CD45, CD3e, F4/80, CD4 or CD8. Data are expressed as means±SEM (*n* = 5).

We have reported previously that Ang II infusion stimulates cardiac inflammation and fibrosis [14], [15], [16], we assessed whether IFN-γ is expressed in the process of Ang II-induced hypertensive cardiac inflammation and fibrosis. Compared with saline-treated mice, Ang II infusion significantly increased IFN-γ positive cells in left-ventricle tissues of WT mice at day 7 after infusion (Figure 1A). The mRNA expression of IFN-γ was also significantly higher in Ang II-treated mice than that of in saline-treated controls at day 1, 3 or 7 after infusion (Figure 1B). To examine if Ang II induces T lymphocytes infiltration into hearts, we used antibodies against CD45 or CD3e (T cell marker) to perform flow cytometry analysis. Flow cytometry analysis revealed that Ang II induced CD45^+^ leukocytes and CD3e^+^ T cells infiltration as early as 1 day of Ang II infusion (Figure 1C). To determine the types of cell producing IFN-γ in Ang II-treated heart, we used antibodies against IFN-γ, F4/80 (macrophage), CD3e (T cell), CD4 or CD8 to perform flow cytometry analysis. Flow cytometry analysis revealed that IFN-γ was primarily produced by CD3e^+^ T cells (both CD4 and CD8 positive cells) in Ang II-treated hearts (Figure 1D), although macrophages could also produce IFN-γ at a lower level.

### Knockout of IFN-γ Reduces Ang II-induced Cardiac Fibrosis

To determine whether IFN-γ has a biological role in Ang II-induced hypertensive cardiac inflammation and remodeling, we used IFN-γ-knockout (IFN-γ KO) mice. After 7-day Ang II infusion, blood pressure increased equally in WT and IFN-γ-KO mice, and baseline systolic blood pressure was also similar in both WT and IFN-γ-KO mice (Table 1, WT sham 101.3±3.9 mmHg vs. WT Ang II 156.2±6.1 mmHg, *p*<0.05; IFN-γ KO sham 110.5±1.6 mmHg vs. IFN-γ KO Ang II 149.8±14.8 mmHg, *p*<0.05). We next assessed the left ventricular (LV) hypertrophy after Ang II-infusion using HW/BW ratios. WT and IFN-γ KO mice had similar ratios under baseline conditions (Table 1). After Ang II infusion for 7 days, there was no significant change in HW/BW ratios in both WT and IFN-γ KO mice (Table 1). We also used echocardiography to evaluate LV size and function, but there was no difference between baseline and Ang II infusion for 7 days (data not shown). These results indicated that short period Ang II infusion (7 days) is not sufficient to cause cardiac hypertrophy.

**Table 1:** Body weight, systolic BP and HW/BW ratios.

	WT	KO	WT+Ang II	KO+Ang II
Parameters	(n = 7)	(n = 7)	(n = 9)	(n = 9)
**BW, g**	23.08±0.47	21.88±0.60	21.85±0.54	19.80±2.49
**HW, mg**	145.00±5.00	130.00±10.00	148.50±4.63	137.60±5.55
**BP, mmHg**	101.34±3.96	110.55±1.65	156.20±6.13*	149.80±14.86*
**HW/BW, mg/g**	6.31±0.52	6.47±0.61	6.78±0.49	7.36±0.67

* *p*<0.05 versus saline-treated group (one-way ANOVA).

Ang II, angiotensin II; BP, blood pressure; BW, body weight; HW, heart weight; WT, wild type; KO, IFN-γ knockout. Data are mean±SEM.

**Figure 2:**
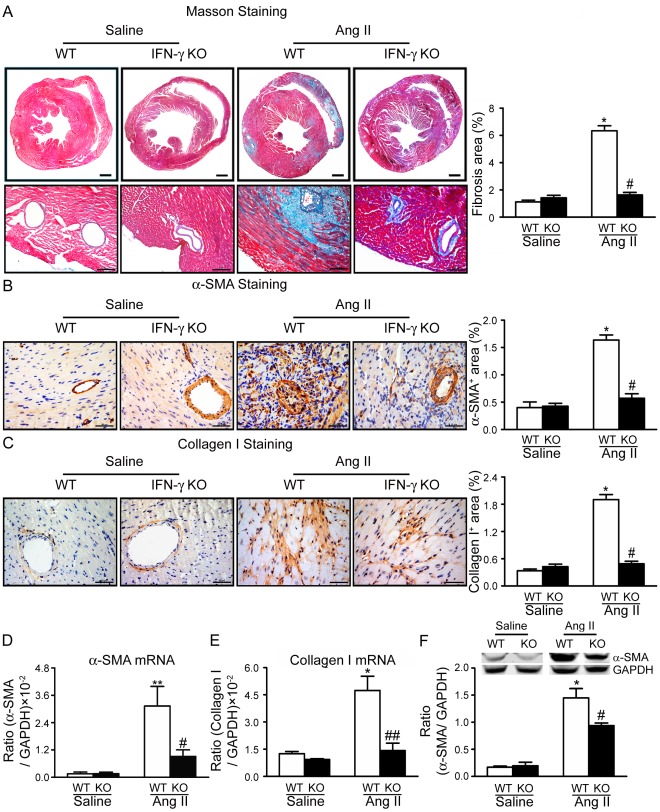
Knockout of IFN-γ reduces Ang II-induced cardiac fibrosis in mouse hearts. **A,** Representative Masson trichrome staining of heart sections from WT and IFN-γ-knockout (IFN-γ-KO) mice (*left panel*). Bar graph shows quantification of fibrotic areas in histological sections (*right panel*; *n* = 8). Magnification: ×40 & ×200. **B–C,** Immunohistochemistry of protein expression of (B) α-smooth muscle actin (α-SMA) and (C) collagen I in WT and IFN-γ-KO hearts. Bar graph shows quantification of areas of positive cells (*right panel*; *n* = 8). Data are mean±SEM. Magnification: ×400. **D,** Real-time PCR analysis of α-SMA mRNA expression (*n* = 5). **E,** Real-time PCR analysis of collagen I mRNA expression (*n* = 5). **F,** Western blots analysis of α-SMA protein levels in WT and IFN-γ-KO hearts with anti-α-SMA antibody. Representative protein bands (*top panel*) and quantitative analysis (*bottom panel*; *n* = 5). * *p*<0.05, **p<0.01 versus saline control; # *p*<0.05, ## *p*<0.01 versus Ang II-infused WT mice.

We then next determined the role of IFN-γ in cardiac fibrosis. Masson’s trichrome staining was performed to evaluate the degree of cardiac fibrosis. Compared with saline-treated mice, Ang-II-treated mice developed cardiac fibrosis (Masson’s trichrome-positive area) at day 7 after infusion (Figure 2A). In contrast, Ang II-induced cardiac fibrosis was significantly reduced in IFN-γ-KO mice. Moreover, knockout of IFN-γ also significantly prevented accumulation of α-SMA-positive cells (a marker of myofibroblast), expression of α-SMA (at both mRNA and protein levels) in cardiac tissues (Figure 2B and 2D and 2F). Finally, knockout of IFN-γ significantly reduced collagen I expression at both mRNA and protein levels (Figure 2C and 2E).

### Knockout of IFN-γ Suppresses Macrophage Infiltration and Downregulates TNF-α Expression in Ang II-treated Hearts

**Figure 3:**
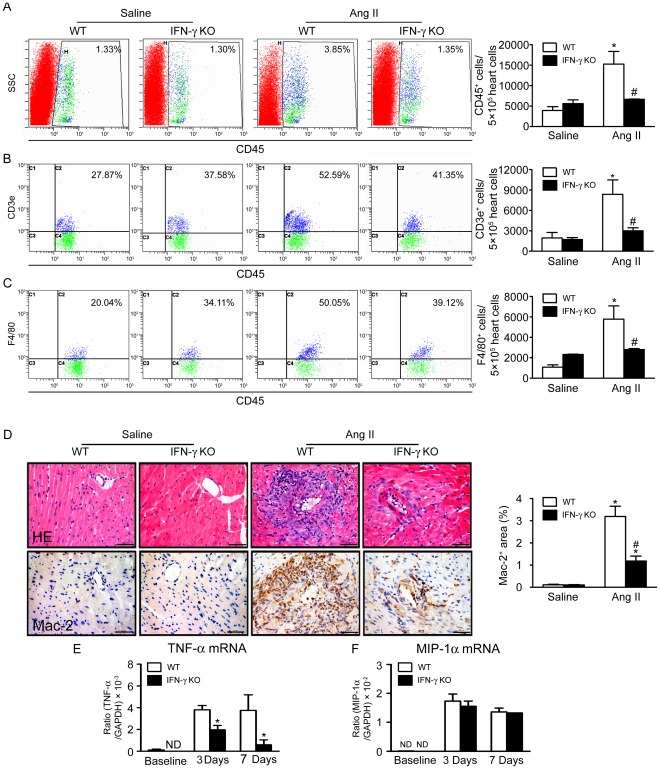
Knockout of IFN-γ suppresses Ang II infusion-induced inflammatory cells infiltration and cytokines expression in hearts. **A–C**, Flow cytometry analysis of (**A**) CD45^+^, (**B**) CD3e^+^, (**C**) F4/80^+^ cells infiltration in Ang II-treated WT and IFN-γ-KO hearts. Bar graph shows quantification of different inflammatory cells in hearts (*n* = 5). **D**, Immunohistochemistry of expression of Mac-2 in WT and IFN-γ-KO hearts with saline or Ang II treatment: hematoxylin and eosin (HE) staining (*top panel*) and antibodies against Mac-2 (*bottom panel*). Bar graph shows quantification of areas of Mac-2^+^ cells. Magnification: ×200. **E–F**, Real-time PCR analysis of (**E**) tumor necrosis factor (TNF)-α Mrna and (**F**) macrophage inflammatory protein (MIP)-1α expression in both WT and IFN-γ KO mice at baseline and day 3, day 7 after Ang II infusion. Data are mean ± SEM (*n* = 5). * *p*<0.05, ** *p*<0.01 versus saline control; # *p*<0.05 versus Ang II-infused WT mice.

To determine how IFN-γ regulates cardiac fibrosis, we measured inflammatory cells infiltration in Ang II-treated WT and IFN-γ-KO hearts by flow cytometry analysis at day 7. Ang-II-treated WT hearts showed an increase in F4/80^+^ macrophages and CD3e^+^ T cells infiltration (Figure 3A–C). Ang II treatment also resulted in an increase in number of Mac-2^+^ macrophages in WT hearts, while the number of Mac-2^+^ macrophages was significantly less in IFN-γ-KO than WT hearts (Figure 3D).

It was reported that peripheral blood T cell secretion of IFN-γ and TNF-α was increased in Ang II-infused WT mice [5]. To examine the effect of IFN-γ on the expression of TNF-α, we evaluated the expression of TNF-α at the mRNA level at baseline and 3 days, 7days after Ang II infusion. Ang II-infusion induced expression of mRNA of TNF-α at early as day 3 and day 7 in WT mice, however, knockout of IFN-γ significantly inhibit the Ang II-induced expression of TNF-α at day 3 and day 7 (Figure 3E). Interestingly, knockout of IFN-γ had not effect of the expression of another cytokine MIP-1α(Figure 3E).

It was reported that cell death may involved in inflammation [17], [18], to evaluate if effect of IFN-γ on Ang-II-induced inflammation and fibrosis is involved in cell death, we performed TUNEL assay in Ang-II-infused hearts. Apoptotic cells were found in the hearts of both WT and IFN-γ KO mice, and there was no significant difference between the two groups (Figure S1A). The cell types of the TUNEL positive cells were analysised by HE staining for serial slide and dual immunofluorescence staining for Troponin I (cardiomyocyte) & TUNEL. Apoptosis was not found in cardiomyocytes after Ang II infusion (Figure S1B). Moreover, we performed serial section analysis of HE staining, the TUNEL positive cells may be primarily in infiltrated inflammatory cells (new Figure S1A). These results indicated that the difference in inflammatory cells infiltration and cytokine expression is not involved in cell death in Ang II-induced inflammation and fibrosis. We next examined how IFN-γ regulates the inflammatory cells infiltration into hearts in response to Ang II infusion.

### IFN-γ Deficiency Decreases MCP-1 Expression In Vivo

**Figure 4:**
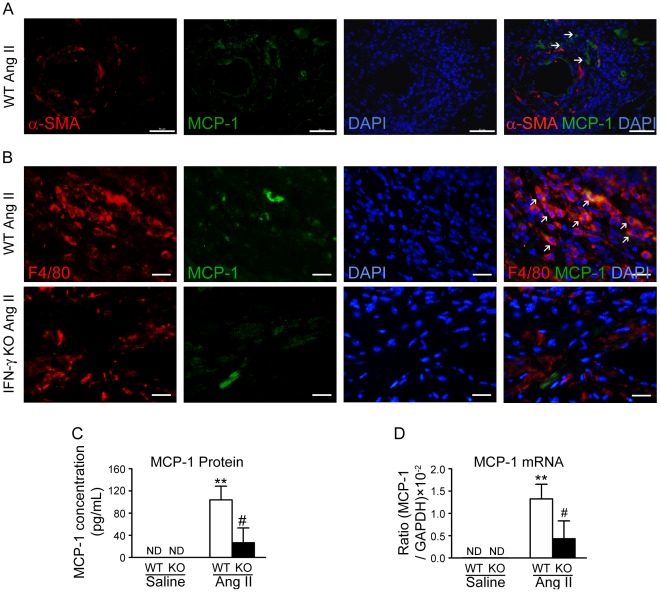
IFN-γ deficiency decreases MCP-1 expression in Ang II-treated mouse hearts. **A,** Immunoflourescence staning for α-SMA (red), MCP-1 (green) and DAPI (blue) in the Ang-II-infused hearts of WT mice. Arrowhead indicate α-SMA**^–^**/MCP-1^+^ cells. **B,** The cryosections of heart were analyzed by double-immunofluorescence staining with antibodies against MCP-1 (green) and F4/80 (red, monocytes/macrophages). DAPI (blue) indicates nuclear staining. Magnification: ×400. **p<0.01 versus saline control; # *p*<0.05 versus Ang II-infused WT mice. **C,** MCP-1 protein level (by CBA) in WT and IFN-γ-KO hearts treated with saline or Ang II for 7 days (*n* = 5). **D,** Real-time PCR quantitative analysis of MCP-1 mRNA expression (*n* = 5).

It has been reported that MCP-1 may be involved in IFN-γ-primed macrophages recruiting leukocytes [19]. Therefore, we determined whether IFN-γ regulates inflammatory cells infiltration by promoting MCP-1 expression in Ang II-induced cardiac fibrosis. To determine the expression of MCP-1 in Ang-II-infused hearts, immunohistochemical staining was performed, MCP-1 was predominantly expressed in α-SMA negative cells around microvessels in Ang-II-infused WT hearts(Figure 4A). To further identify the types of cell population responsible for the MCP-1 production in Ang II-infused hearts, we performed double-immunofluorescence staining using antibodies against MCP-1 and F4/80 (macrophage). We found that MCP-1 was primarily expressed in macrophages in Ang II treated-hearts in WT mice while the number of MCP-1 positive cells was significantly less in IFN-γ KO mice (Figure 4B). We next determined the MCP-1 expression at protein and mRNA levels in heart tissue by CBA Flex set assays and quantitative real-time PCR. As shown in Figure 4C&D, the Ang II-induced increase in MCP-1 expression in WT mice was significantly reduced in IFN-γ-KO mice. Therefore, in the absence of IFN-γ, MCP-1 expression is impaired and this may be responsible for reduced leukocytes infiltrating into cardiac tissues.

### Macrophage Stimulates Expression of IFN-γ in T cells That Is Essential for Macrophage Migration

**Figure 5:**
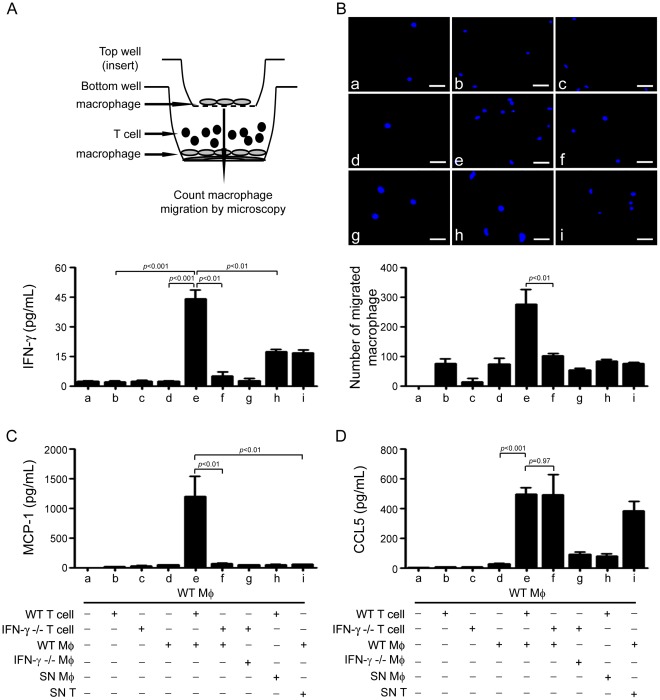
Crosstalk between IFN-γ-producing T cells and macrophages augments IFN-γ and MCP-1 expression and macrophage migration. **A**, IFN-γ expression was determined by incubating T cells either with macrophages isolated from and WT or IFN-γ-KO mice or with the supernatant of macrophages. Bar graph represents the quantification of IFN-γ level in the supernatant of the cell culture. **B**, Transwell assay of macrophages migration. WT macrophages were plated on the upper chambers of transwell inserts. The lower chamber was consisted of co-cultured WT or IFN-γ-KO T cells with WT macrophage (as described in **A**, *upper panel*). Macrophages that had migrated to the lower chamber were counted by DAPI staining. Bar graph represents the cell numbers that migrated into the lower chamber. **C–D**, Quantification of (C) MCP-1 or (D) CCL5 levels in the supernatant of the cell cultures as described in **A**. * *p*<0.05, **p<0.01 versus co-culture of IFN-γ-KO T cells with WT macrophages. Mφ, macrophage.

As we showed IFN-γ was primarily expressed in T cells, we next to determine how IFN-γ expression is regulated in T cells. Macrophages and T cells were isolated from WT or IFN-γ KO mice, and used for co-culture experiments. As shown in Figure 5A, T cells or macrophages alone did not express IFN-γ, however, co-culture of WT T cells and macrophages significantly increased IFN-γ expression (19.8±0.95 folds, vs. WT macrophage, *p*<0.001; 20.9±2.09 folds, vs. WT T cells, *p*<0.001). Co-culture of IFN-γ-KO T cells with WT macrophages produced significantly lower level of IFN-γ ( *p*<0.01). These results indicate that IFN-γ is produced by T cells and this process requires macrophages. To investigate whether macrophage promotes the IFN-γ production of T cells by directly acting on T cells or by secreting soluble mediators, we incubated T cells either with the supernatant of WT macrophages (SN Mφ) or with WT macrophages (Figure 5A). Compared with T cells coculture with WT macrophages, SN Mφ produced much lower than IFN-γ secretion in T cells, suggesting direct cell interaction between macrophages and T cells promotes the expression of IFN-γ.

To determine the role of IFN-γ in inflammatory cells infiltration, we performed transwell migration assays (Figure 5B, up panel) to investigate if IFN-γ deficiency reduces macrophage infiltration. As shown in Figure 5B, T cells or macrophages alone did not stimulated macrophage migration. Co-culture of WT T cells with WT macrophages increased macrophage migration significantly (2.5±0.8 folds, co-culture of WT T cells and macrophage vs. co-culture of IFN-γ-KO T cells and macrophage, *p*<0.01), however co-culture of IFN-γ-KO T cells with WT macrophages failed to stimulate macrophage migration**.**

### IFN-γ in T Cells Augments the Expression of MCP-1

Because MCP-1 production was decreased in IFN-γ-KO mice, we therefore determined if IFN-γ-producing-T cells regulate chemokines expression that stimulates macrophage migration, we measured the chemokines production in the co-culture of T cells with macrophages. Similar to the results found *in vivo*, MCP-1 production, but not CCL5 production was significantly increased in co-culture of WT T cells with WT macrophages (Figure 5C and 5D). In co-culture of IFN-γ-KO T cells and WT macrophages, MCP-1 production was significantly lower than that in co-culture of WT T cells and WT macrophages (95.8±1.2%, *p*<0.05) (Figure 5C). MCP-1 production was decreased significantly when T cells and macrophages were separated or incubating purified T cells with SN Mφ (Figure 5C, column b, d, h). Whereas WT macrophage inbutated with SN T probably produced more CCL5, indicating that some factors derived from T cells acting on macrophage to promote the CCL5 production (Figure 5D, column i). Since T cells express AT-1R, we test if Ang II directly stimulate IFN-γ expression in T cells, thymus T cells were treated with Ang II (100 nM) for 24 hrs, and mRNA of IFN-γ was measured by RT-PCR. As shown in new Figure S3, Ang II treatment did not significantly increased the expression of IFN-γ compared to that of the untreated T cells.

## Discussion

The inflammatory response plays a critical role in hypertension-induced cardiac remodeling; however, how inflammatory responses are activated and their specific roles in cardiac remodeling remain unclear. We showed that knockout of IFN-γ significantly reduced Ang II infusion-induced inflammation and cardiac fibrosis in mice. T cells infiltration in mouse hearts was a major source of IFN-γ production, and contact-mediated effect between IFN-γ-producing T cells and macrophage stimulated macrophages production of MCP-1, which recruited more macrophages and fueled an inflammatory-positive feedback loop.

Ang II can affect the immune responses by amplifying the expression of cytokines and chemokines in macrophages, regulating dendritic cell differentiation, and promoting lymphocyte proliferation [20]. Inflammation is regulated by the presence of immune cells such as T cells and macrophages and released inflammatory mediators such as cytokines and chemokines. Cytokines are critical regulators of immunity and inflammation and regulate stages of hypertension [5], [21]. Cytokines such as IL-1, IL-6, IL-10, IFN-γ, TNF-α or TGF-β have high expression in Ang II-treated vascular systems and exhibit pro- and anti-fibrosis actions [21], [22]. Ang II-induced fibrosis in the heart and kidneys is mediated by blood pressure and calcineurin-dependent pathways [23]. We demonstrated that Ang II infusion rapidly induced the expression of the cytokine IFN-γ in hearts (Figure 1). Moreover, we found there is no IFN-γ expression in cardiomyocytes and fibroblasts (data not shown), our result is consistent with previous studies that has documented the production of IFN-γ is primarily by T cells and natural killer (NK) cells, possibly by antigen-presenting cells (e.g. macrophages and dendritic cells) in response to IL-12 but not other type of cells [24], [25], [26].

Fairweather et al reported that IFN-γ deficiency increased chronic myocarditis, pericarditis and fibrosis after CB3 virus infection. Interestingly, they also found that IFN-γ deficiency didn’t significantly alter myocardial inflammation during acute myocarditis at day 12 after CB3 infection, although which reduced viral replication in the heart [27], [28]. In contrast to infection, studies of an Ang II-accelerated atherosclerotic model have suggested IFN-γ is a key factor in the pathogenesis of atherosclerosis [29]. Our results indicated that IFN-γ has a key role in the progression of Ang II-induced cardiac inflammation and fibrosis (Figure 2 & 3). Although knockout of IFN-γ did not prevent the increase in systolic blood pressure after Ang II infusion (Table 1), it prevented acute Ang II-induced inflammation (day 7 after infusion) such as macrophages and T cells infiltration in cardiac tissues and expression of TNF-α (Figure 3). In agreement with our results, neutralization of IFN-γ during challenge with antigen plus IL-18 inhibited the combination of eoainophilic infiltration, lung fibrosis, and periostin deposition or the combination of neutrophilic infiltration and airway hyperresponsiveness, respectively [12].

Our results showed that T cells accumulated and produced IFN-γ in the mouse hearts with Ang II infusion (Figure 1C–D & 3A–B). Furthermore, the contact-mediated effect between macrophages and T cells stimulated MCP-1 production and macrophage migration in WT mice (Figure 5), which was reduced in IFN-γ-KO mice (Figure 4). We demonstrated that IFN-γ-producing T cells is essential for the infiltration of macrophages in Ang II-treated hearts (Figure 5). It is known that T cells play a major role in mediating inflammatory disorders by contributing to or causing tissue damage through the release of IFN-γ. Several reports showed that CD4^+^ T cells producing IFN-γ controlled the differentiation, migration and activation of macrophage lineage cells in myocarditis, central nervous system and Ang II-induced kidney injury [30], [31], [32]. CD8^+^ T cells and adipose tissue interact with each other to recruit macrophages and activate a local inflammatory cascade to mediate aortic aneurysm [33].

We found MCP-1 expression was increased in Ang II-treated hearts, and when IFN-γ is deleted, MCP-1 production was significantly reduced (Figure 4 & 5C). Previous works from others and us demonstrated that MCP-1 is necessary to induce Ang II-mediated fibrosis in the myocardium [14], [34], which expression in macrophage, endothelium, vascular smooth muscle cells and glomerular endothelial and epithelial cells [35], [36]. We found that WT macrophages interacting directly with WT T cells stimulated more MCP-1 production than WT macrophages interacting with IFN-γ-KO T cells (Figure 5C), providing an explanation of how IFN-γ is responsible for amplifying the expression of MCP-1. Therefore, our findings established an interaction between T cell produced IFN-γ and macrophage production of MCP-1. Lin et al used transgenic mice, called “macrophages insensitive to interferon-γ” mice to assess the effects of IFN-γ signaling on macrophage lineage cells in response to infection of lymphocytic choriomeningitis virus, they reported that CD4^+^ T-cell production of IFN-γ promotes signaling in macrophage lineage cells, which control the production of chemokines i.e., MCP-1, and the recruitment of macrophages to the central neuron system [31]. Coelho et al reported that priming of macrophage with IFN-γ stimulated production of MCP-1, which may drive tissue chemokine production and inflammation and bear a significant role in the pathogenesis of Chagas disease [19]. When this manuscript is under review, Pore et al reported that a similar observation of that coincubation with outer membrane protein A of Shigella flexneri 2a -pretreated macrophages enhances the production of IFN-γ by the outer membrane protein A-primed CD4^+^ T cells, representing that outer membrane protein A may enhance IFN-γ expression in CD4^+^ T cells through the induction of IL-12 production in macrophages. They demonstrated that TLR2 activation and antigen presentation is responsible for the optimum production of IFN-γ by macrophages:CD4^+^ T cells coculture [37].

We found that another CC-chemokine–macrophage inflammatory protein 1α (MIP-1α)–expression did not differ between WT and IFN-γ KO mice at day 7 after Ang II-infusion (Figure 3E). Although MCP-1 and MIP-α are belong to CC chemokine family, Natasa et al revealed that MCP-1 expression was unregulated significantly in patients of intermediate uveitis (IU) and there is no difference in MIP-1α between IU and control patients in intraocular levels [38].

**Figure 6:**
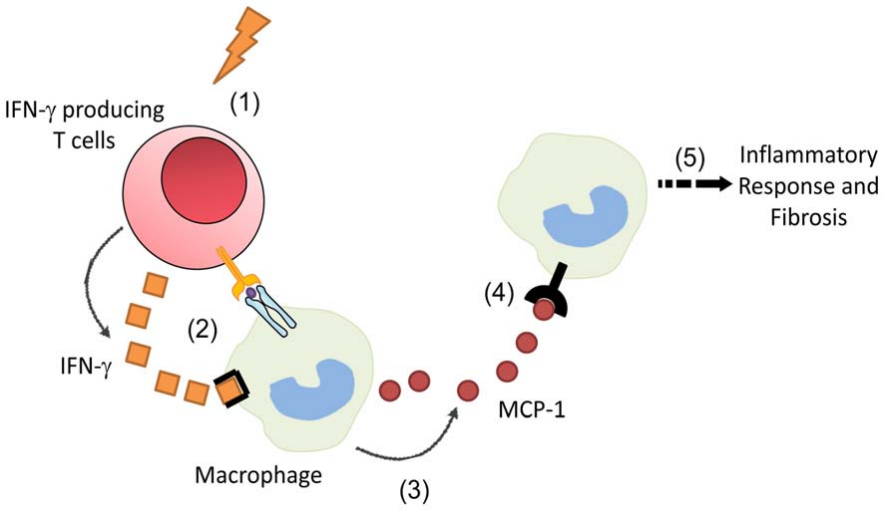
Summarize of the role of IFN-γ in angiotensin II-induced cardiac fibrosis and inflammation. Hypertension or Ang II induce the infiltration of IFN-γ producing T cells (1); that interact directly with macrophages and release IFN-γ (2); which activates marcrophages and increases the production of MCP-1 (3); which recruits more macrophages to heart (4); then fuels inflammation and fibrosis (5).

Our results reveal an important role of IFN-γ in Ang II-infusion-induced inflammation and cardiac fibrosis, which is summarized in Figure 6. IFN-γ is essential for inflammatory cell infiltration, expression of pro-inflammatory cytokines, and cardiac fibrosis in Ang II-treated mouse hearts. Our results demonstrate that there is a reciprocal interaction between infiltrated T cells and macrophages in heart where IFN-γ produced by T cells stimulates the macrophage expression of MCP-1, which causes the recruitment of macrophages and leukocytes and cardiac inflammation and fibrosis.

## Materials and Methods

### Animals and Ethics Statement

B6.129S7-Ifng^tm1Ts^/J (IFN-γ^−/−^) mice and wild-type littermates were purchased from the Jackson Laboratory [39]. Mice were maintained under specific-pathogen-free conditions in the animal facility at the Beijing Heart Lung and Blood Vessel Diseases Institute. The mice were given a standard diet. The investigations conformed to the US National Institutes of Health Guide for the Care and Use of Laboratory Animals (publication no. 85–23, 1996) and were approved by the Animal Care and Use Committee of Capital Medical University.

### Mouse Model of Ang II Infusion-induced Cardiac Fibrosis

Hypertensive cardiac fibrosis was induced in 6–8 week old IFN-γ-knockout (IFN-γ–KO) mice and littermate wild-type (WT) mice by subcutaneous infusion of Ang II (Sigma-Aldrich St. Louis, MO) at a dose of 1500 ng/kg/minute using osmotic minipumps (Alzet MODEL 1007D; DURECT, Cupertino, CA) as we described before [14]. We also infused mice for different days to investigate the time course effect. Systolic blood pressure was measured by the tail-cuff system (Softron BP-98A; Softron, Tokyo, Japan), and values were derived from a mean of 10–20 measurements per animal at each time point. All animals were euthanized by overdose pentobarbital (100 mg/kg) at the end of each treatment period.

### Histopathology and Immunohistochemistry

Hearts from WT and IFN-γ mice fixed in 10% formalin were processed and paraffin embedded. Heart sections (4 µm) were then stained with hematoxylin and eosin (H & E) and Masson’s trichrome reagent [40]. The percent of fibrosis (blue staining) to total tissue was analyzed and calculated by a NIS-ELEMENTS quantitative automatic program (Nikon, Tokyo, Japan) with the average value of at least 8 images per heart in double-blind fashion**.** Serial, transverse cryosections (7 µm thick) of hearts were cut by use of a CM1950 Frigocut (Leica, Wetzlar, Germany) at −20°C and were kept at −80°C.

Immunohistochemical staining was performed as described [41]. Heart sections were stained with anti-rabbit antibodies against Mac-2 (1∶500 dilution, Santa Cruz Biotechnology, Santa Cruz, CA), collagen I (1∶1000), and α-smooth muscle actin (α-SMA, 1∶200; all Abcam, Cambridge, MA), and rabbit IgG or rabbit serum instead of primary antibody was used as negative control (Figure S2). Peroxidase activity was visualized with use of diaminobenzidine, and sections were counterstained with hematoxylin. Images were obtained by the use of a CCD camera under a microscope (ECLIPSE80i/90i, Nikon, Japan) with a ×200 lens, and 10–20 fields/section were chosen randomly. Cryosections (7 µm) of hearts were stained with anti-IFN-γ (1∶50 dilution, Abcam), anti-MCP-1 (1∶100 dilution, Santa Cruz), anti-F4/80 (1∶100 dilution, Abcam) and anti-Troponin I (1∶100 dilution, Santa Cruz) specific antibodies, followed by IgG specific, peroxidase- or fluorescence-conjugated secondary antibodies as described. For each mouse, 8 slices (4 apical and 4 proximal to the aortic valve on septum and free wall) were analyzed in double-blind fashion.

### TUNEL Staining

The TUNEL procedure was performed with the In Situ Apoptosis Kit (Promega, Madison, MI). Five micron thick frozen sections are cut and airdried at 4°C for 48 hours. Following a cold acetone fixation for 10 minutes, the slides are airdried for 2 minutes before 3 rinses in PBS. The slides are then rinsed in 1× TdT buffer for 5 minutes before careful application of the TdT reation mix to the tissue sections on each slide, which are then incubated in a humid chamber at 37°C for 60 minutes, followed by 3 PBS washes. The slides are then stained with DAPI and coverslipped using Vectashield for viewing on the Nikon epifluorescent microscope.

### Flow Cytometry

Hearts were harvested and cardiac cell suspensions were prepared as described [42]. Briefly, hearts were harvested, then minced cardiac tissue was digested with 0.1% collagenase B and 2.4 U/mL dispase II (both Roche Molecular Biochemicals) at 37°C for 30 min. The dissolved tissue was then passed through a 70 µm sterile filter (Falcon, BD, Franklin Lakes, NJ) yielding a single cell suspension. Cells were washed twice with Hanks’ balanced salt solution (HBSS) buffer with 2% FBS, then underwent cell-surface and intra-cellular antigen staining with fluorochrome-conjugated monoclonal rat anti-mouse antibodies against CD45, CD3e, CD4, CD8, F4/80 or IFN-γ (all BioLegend, San Diego, CA) at 4°C for 30 min.

Flow cytometry was used to characterize the infiltration of T cells and macrophages into hearts of saline and Ang II–treated mice and the cell source of IFN-γ involved by use of Epics XL equipment (Beckman Coulter, Miami, FL). Data were analyzed by use of Summit software (Beckman Coulter).

### Real-time PCR

Total ventricular RNA was extracted by use of TRIzol reagent (Invitrogen, Carlsbad, CA) according to the manufacturer’s protocol. PCR amplification involved use of the iQ5 Real-Time PCR Detection System (Bio-Rad, Hercules, CA) with SYBR Green JumpStart^TM^ Taq ReadyMix^TM^ (Takara, Otsu, Shiga, Japan) and primers for mouse IFN-γ, Collagen I, α-SMA, TNF-α, MCP-1, MIP-1α and GAPDH. Melting curve analysis was performed at the end of each PCR reaction**.** The housekeeping gene GAPDH was used as control: the expression of those genes was expressed as a ratio to that of GAPDH. Primer sequences were as follows: Collagen I, forward 5′-GAGCGGAGAGTACTGGATCG-3′ and reverse 5′-TACTCGAACGGGAATCCATC-3′; MCP-1, forward 5′-GTCTGTGCTGACCCCAAGAAG-3′ and reverse 5′- TGGTTCCGATCCAGGTTTTTA-3′; MIP-1α, forward 5′-GCTGACAAGCTCA CCCTCTGT-3′ and reverse 5′-GGCAGTGGTGGAGACCTTCA-3′; IFN-γ, forward 5′-TGCTGATGGGAGGAGATGTCT-3′ and reverse 5′- TTTCTTTCAGGGACAGCCTGTT-3′; and α-SMA, forward 5′- CCCACCCAGAGTGGAGAA-3′ and reverse 5′-ACATAGCTGGAGCAGCGTCT-3′;and GAPDH, forward 5′-CCTGGAGAAACCTGCCAAGTATGA-3′ and reverse 5′-TTGAAGTCACAGGAGACAACCTGG-3′.

### Western Blot Analysis

Heart tissues were harvested at the end of each treatment period, immediately frozen in liquid nitrogen, and then homogenized in lysis buffer. Western blot analysis was performed as described [21].

### Cytokine Measurements

To analyze the effect of Ang II on cytokine and chemokine production in hearts, heart tissue were harvested at the end of experiment, immediately frozen in liquid nitrogen, and then homogenized in lysis buffer. After centrifugation, the supernatants of tissue were collected and analyzed using the Cytometric Bead Array Flex Set system (BD Biosciences, San Jose, CA) to measure secreted MCP-1. To measure the concentration of IFN-γ, MCP-1, and CCL5 in cell culture media, 50 µL supernatant from each sample was incubated with the CBA Flex Set beads assay for 2 hr. The fluorescence produced by the beads was measured on a FACS Calibur flow cytometer (BD Biosciences) and analyzed by the associated software.

### Isolation of Naîve T Cells and Macrophages

The thymus and attached blood vessels were removed from 6- to 8-week-old mice and washed in phosphate buffered saline (PBS) and ground by use of frosted glass in RPMI 1640 medium (HyClone; Thermo Fisher Scientific, Waltham, MA). Suspensions were cleared of connective tissue by filtration and then underwent Ficoll-gradient centrifugation (HaoYang, TianJin, China) to clear residual erythrocytes and non-lymphocytes. T cells were cultured in RPMI 1640 medium containing 10% fetal bovine serum (FBS), 1 µg/mL anti-CD3e (eBioscience, San Diego, CA), 1 µg/mL anti-CD28 (BioLegend, San Diego, CA), penicillin and streptomycin.

Macrophages were isolated from bone marrow of mice and grown in macrophage colony-stimulating factor (M-CSF; PeproTech, Rocky Hill, NJ) as we described [43] with minor modification. Briefly, bone-marrow cells were isolated from femurs and tibias of 8- to 12-week-old mice. Suspensions were cleared of adipose tissue and connective tissue by filtration and then underwent Ficoll-gradient centrifugation to clear residual erythrocytes and non-lymphocytes. Myeloid origin macrophage were cultured in DMEM medium (HyClone, Waltham, MA) supplemented with 10% heat-inactivated FBS in the presence of 50 ng/mL M-CSF.

### *In Vitro* Migration Assay

Cell migration was quantitated in duplicate by use of 24-well Transwell inserts with polycarbonate filters (8-µm pore size) (Corning Costar, Acon, MA). Macrophage (2.5×10^3^ in 250 µL DMEM high-glucose medium/10% FBS) was added to the upper chamber of the insert. The lower chamber contained macrophage (1.0×10^5^) and/or activated T cells (1.0×10^6^) in 1 mL RPMI 1640 medium/10% FBS isolated from WT and IFN-γ–KO mice. The plates were incubated at 37°C in 5% CO_2_ for 12 hr. Cells that had migrated were counted by use of DAPI staining.

### Statistical Analysis

All data are expressed as the means±the SEM. The unpaired 2-tailed *t*-test was used to compare the 2 groups. Comparisons between the wild-type (WT) group and IFN-γ KO group were performed using one-way ANOVA by Newman-Keuls multiple comparison test from GraphPad Prism (GraphPad Software). When any significant difference (*p*<0.05) was seen in the main effect (group differences), the comparison was analyzed by the unpaired 2-tailed *t*-test. For the other comparisons, we determined the significance of difference between the means of the groups by one-way ANOVA. The difference was considered statistically significant at *p*<0.05.

## Supporting Information

Figure S1 **TUNEL assay in Ang-II-infused hearts. A.** Apoptic cells were found in the hearts of both WT and IFN-γ KO mice. At 7 days after Ang II infusion, serial slides of the hearts were examined. Apoptotic positive cells were stained by TUNEL staining; The cell types of the TUNEL positive cells were analysised by HE staining. Bar graph shows semi-quantification of ratio of TUNEL^+^ cells to total cells. Arrows indicate positive TUNEL staining cells. Magnification: ×200. **B.** Apoptosis was not found in cardiomyocytes after Ang II infusion. Dual immunofluorescence staining for Troponin I (red, cardiomyocyte), TUNEL (green) and DAPI (blue, nuclei). Arrows indicate positive TUNEL staining cells. Magnification: ×400.
(TIF)

Figure S2 **Negative antibody was replaced by rabbit IgG.** Heart sections were stained with anti-rabbit antibodies against Mac-2, collagen I, and α-SMA, and rabbit IgG or rabbit serum instead of primary antibody was used as negative control. Magnification: ×400.
(TIF)

Figure S3 **No difference of expression of IFN-γ between Ang II-treated T cells and untreated T cells.** Thymus T cells were treated with Ang II (100 nM) for 24 hr, the mRNA of IFN-γ was measured by RT-PCR. Bar graph show that Ang II treatment did not significantly increased compared to that of the untreated T cells.
(TIF)
