# Supplementary figures and images for: Reciprocal Interaction between Macrophages and T cells Stimulates IFN-γ and MCP-1 Production in Ang II-induced Cardiac Inflammation and Fibrosis
Source: PLoS One. 2012 May 2;7(5):e35506. doi: 10.1371/journal.pone.0035506 (PMC3342394; doi:10.1371/journal.pone.0035506)

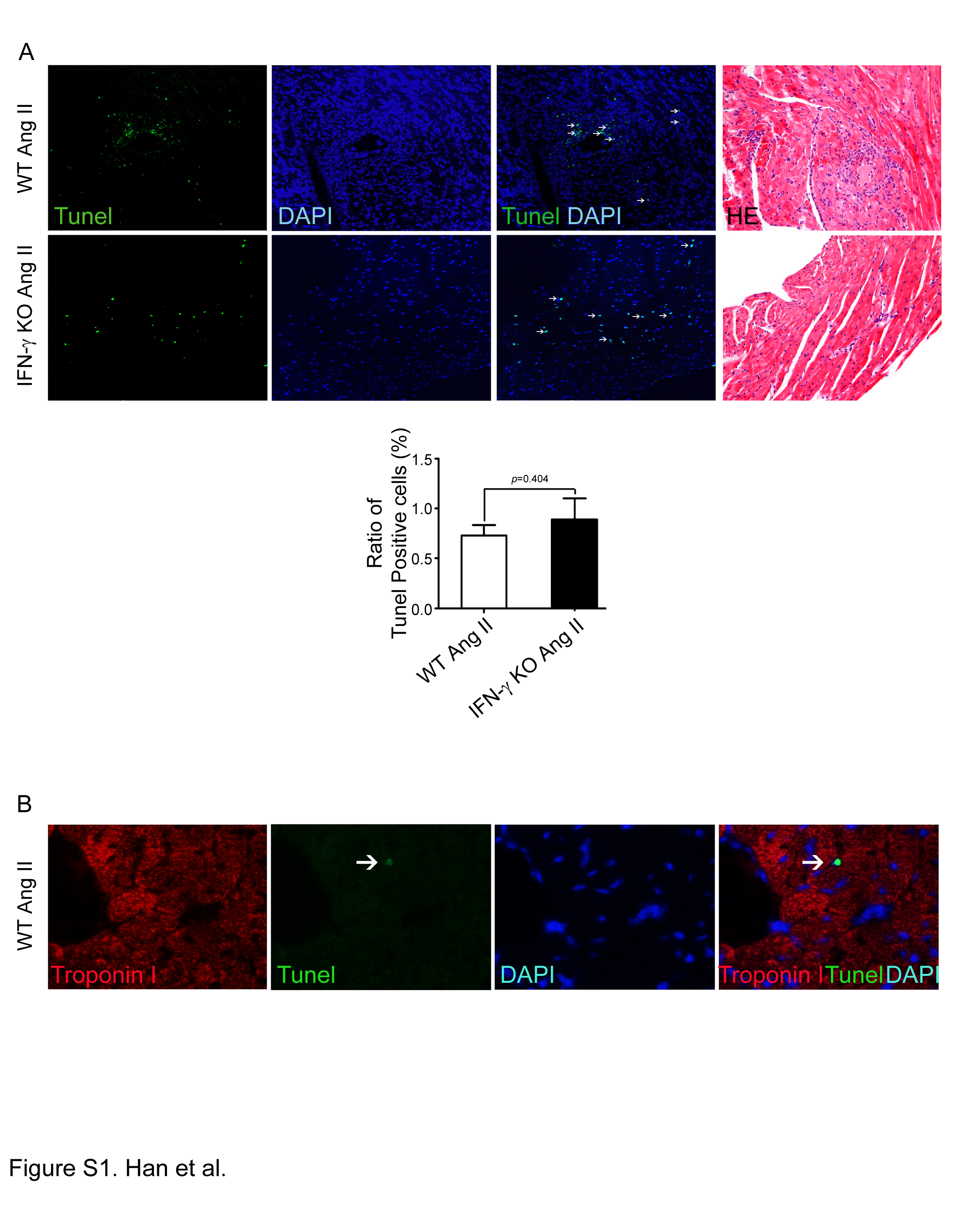

Supplement: Figure S1 — TUNEL assay in Ang-II-infused hearts. A. Apoptic cells were found in the hearts of both WT and IFN-γ KO mice. At 7 days after Ang II infusion, serial slides of the hearts were examined. Apoptotic positive cells were stained by TUNEL staining; The cell types of the TUNEL positive cells were analysised by HE staining. Bar graph shows semi-quantification of ratio of TUNEL+ cells to total cells. Arrows indicate positive TUNEL staining cells. Magnification: ×200. B. Apoptosis was not found in cardiomyocytes after Ang II infusion. Dual immunofluorescence staining for Troponin I (red, cardiomyocyte), TUNEL (green) and DAPI (blue, nuclei). Arrows indicate positive TUNEL staining cells. Magnification: ×400. (TIF) [file pone.0035506.s001.tif]

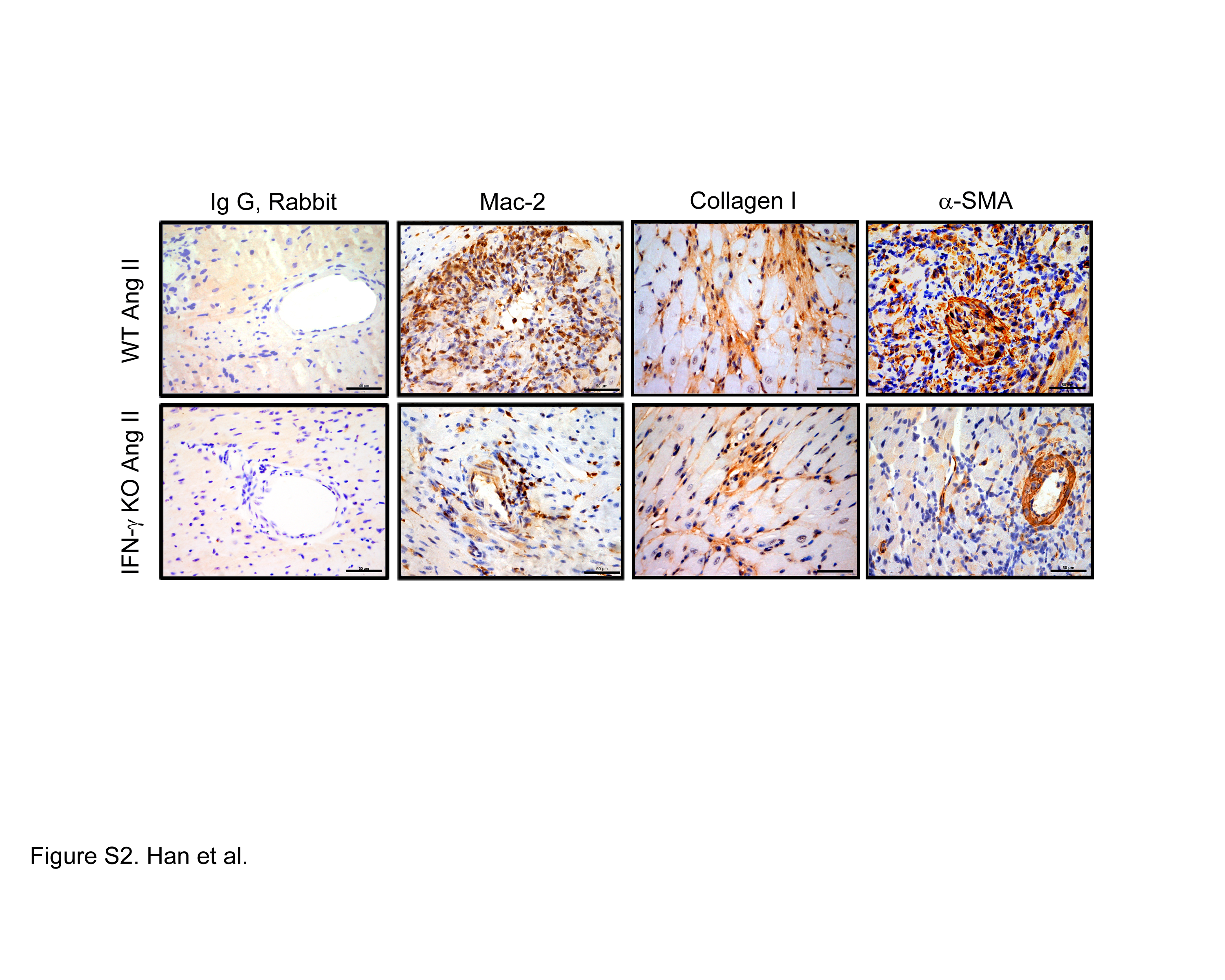

Supplement: Figure S2 — Negative antibody was replaced by rabbit IgG. Heart sections were stained with anti-rabbit antibodies against Mac-2, collagen I, and α-SMA, and rabbit IgG or rabbit serum instead of primary antibody was used as negative control. Magnification: ×400. (TIF) [file pone.0035506.s002.tif]

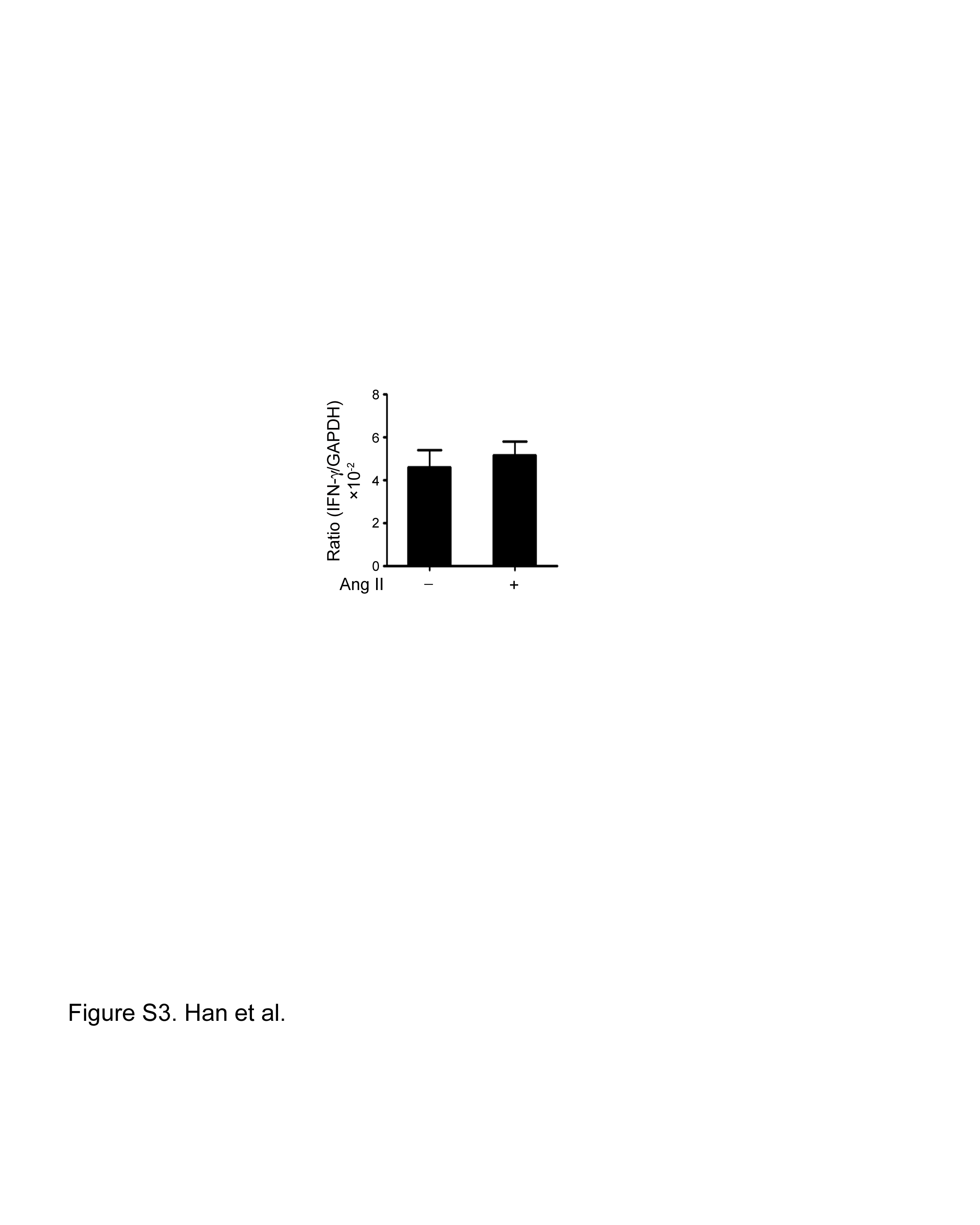

Supplement: Figure S3 — No difference of expression of IFN-γ between Ang II-treated T cells and untreated T cells. Thymus T cells were treated with Ang II (100 nM) for 24 hr, the mRNA of IFN-γ was measured by RT-PCR. Bar graph show that Ang II treatment did not significantly increased compared to that of the untreated T cells. (TIF) [file pone.0035506.s003.tif]
